# False positive reduction in protein-protein interaction predictions using gene ontology annotations

**DOI:** 10.1186/1471-2105-8-262

**Published:** 2007-07-23

**Authors:** Mahmoud A Mahdavi, Yen-Han Lin

**Affiliations:** 1 Department of Chemical Engineering, University of Saskatchewan, 57 Campus Drive, Saskatoon, SK, S7N 5A9, Canada

## Abstract

**Background:**

Many crucial cellular operations such as metabolism, signalling, and regulations are based on protein-protein interactions. However, the lack of robust protein-protein interaction information is a challenge. One reason for the lack of solid protein-protein interaction information is poor agreement between experimental findings and computational sets that, in turn, comes from huge false positive predictions in computational approaches. Reduction of false positive predictions and enhancing true positive fraction of computationally predicted protein-protein interaction datasets based on highly confident experimental results has not been adequately investigated.

**Results:**

Gene Ontology (GO) annotations were used to reduce false positive protein-protein interactions (PPI) pairs resulting from computational predictions. Using experimentally obtained PPI pairs as a training dataset, eight top-ranking keywords were extracted from GO molecular function annotations. The sensitivity of these keywords is 64.21% in the yeast experimental dataset and 80.83% in the worm experimental dataset. The specificities, a measure of recovery power, of these keywords applied to four predicted PPI datasets for each studied organisms, are 48.32% and 46.49% (by average of four datasets) in yeast and worm, respectively. Based on eight top-ranking keywords and co-localization of interacting proteins a set of two knowledge rules were deduced and applied to remove false positive protein pairs. The '*strength*', a measure of improvement provided by the rules was defined based on the signal-to-noise ratio and implemented to measure the applicability of knowledge rules applying to the predicted PPI datasets. Depending on the employed PPI-predicting methods, the *strength *varies between two and ten-fold of randomly removing protein pairs from the datasets.

**Conclusion:**

Gene Ontology annotations along with the deduced knowledge rules could be implemented to partially remove false predicted PPI pairs. Removal of false positives from predicted datasets increases the true positive fractions of the datasets and improves the robustness of predicted pairs as compared to random protein pairing, and eventually results in better overlap with experimental results.

## Background

In recent years high throughput technologies have provided experimental tools to identify protein interactions in large scale, generating tremendous amount of protein interaction data [1]. On the other hand, however, computational approaches for protein interaction inference have presented a growing number of inexpensive methods to predict vast number of protein pairs on genome scale [2]. Both experimental techniques and computational approaches are affected by high false positives and false negatives that tend to result in poor agreement among bench mark datasets [3]. On the experimental front, false positives mostly stem from the technology involved. Nonetheless, some techniques have been already proposed to enhance the reliability of current high-throughput screening datasets [4]. On the computational front, most efforts have been focused on predicting more protein-protein interactions by means of various approaches identifying true positives that bring along numerous false positive and false negative predictions. Reduction of false positive predictions in computational approaches has not been adequately investigated.

So far, several computational approaches have been proposed to predict protein interactions [5]. These approaches can be grouped into six categories based upon the hypotheses from which they originate. The first category comprises the methods that utilize genomics information to predict protein interactions. Conventional phylogenetic profiles [6], gene fusion [7], gene neighbourhood [8], and transgenic distance [9] are as such. Availability of whole genome sequences has enabled the genome scale homology search, resulting in the construction of protein profiles, discovery of fusion events, specifying gene orders, and identifying genetic distances in the above-mentioned genome-based methods. In second category are the methods that rely on statistical scoring functions such as mutual information [10], Jaccard coefficient [11], and chance co-occurrence distribution [12] to calculate the extent of similarity of protein phylogenetic profiles. These methods have been recently employed to enrich conventional genomics methods by using score functions instead of exact similarity of patterns or genetic localizations. The third group is domain-based protein interaction prediction methods. These methods postulate that conservation of sequence properties such as domains, motifs, and signatures over the course of evolution may contribute to the interaction of proteins. Earlier studies focus on an association method [13] which was improved by considering the number of signatures in each protein sequence [14]. The likelihood approach was also implemented to enhance the feasibility of domain-based approaches [15]. The fourth category consists of the methods in which structural similarities and prediction upon structural models is the underlying hypothesis. These methods range from the threading approach [16], docking methods [17], and the CAPRI experiment [18] to protein interaction prediction based on surface patch comparison [19] and oligomeric protein structure networks [20]. The fifth category covers the methods which employ machine learning techniques to predict protein-protein interactions. These methods use different information to predict protein-protein interactions such as primary structures [21], and conserved network motifs [22]. Interaction mining was also used to train learning systems to recognize correlated patterns within protein interaction pairs [23]. Support vector machines (SVM) have been used to construct supervised classifiers in order to identify interacting proteins [24]. The effect of the training dataset on the performance of SVM prediction has been studied [25] to enhance the efficiency of predictions. The sixth category includes the methods that use gene co-expression information to predict protein-protein interactions [26]. These methods predict interacting proteins through integration of micro array data in different biological conditions and construction of co-expression profiles for genes [27].

False positive prediction in all computational methods and their limited overlap with experimental results are post-genomic challenges. Computational PPI prediction approaches consider protein-protein interactions in the most general context and often refer to 'functionally interacting proteins', implying that the proteins cooperate to perform a given task without necessarily involving any physical contact. Experimental PPI detection techniques, such as yeast two-hybrid and large-scale affinity purification with mass spectrometry, attempt to discover direct physical interactions between proteins. However, there is a limited overlap between sets of interacting proteins identified by functional and physical relationships [28]. Given the incomplete coverage of experimental results, there is clearly the need to develop large-scale robust computational sets of interacting proteins validated by future experiments. Furthermore, because of the lack of solid information on protein-protein interaction, the accuracy of different computational approaches remains uncertain. Nevertheless, it is a common perception that if both experimental results and computational predictions agree on a link, the confidence level of that link would be high. Therefore, one measure to evaluate the false positive content of computational predictions is the level of agreement with experimental findings. Although high-throughput screening techniques are affected by false positives, validation of computational pairs by experimental results is widely acceptable.

To enhance the overlap between computational predictions and experimental results, we need to find a systematic way to remove false positives, resulting in an increased true positive fraction of every predicted PPI dataset. In order to achieve this goal a common ground upon which the predicted results can be evaluated is required. Gene Ontology (GO) annotations may serve as the common ground, even though annotation is an ongoing process. Gene Ontology (GO) is the database that contains controlled vocabularies to annotate molecular attributes for different model organisms. Annotations are defined in three structured ontologies which allow the description of molecular function (F), biological process (P), and cellular component (C). Each ontology is structured in child-parent hierarchies in which a 'child' may have many 'parents' and child terms are components of parent terms. Thus, information provided by GO must be useful in further assessment of predicted PPIs and may be integrated with global filtering algorithms to reduce the number of false positives in PPI predicted datasets. Currently, several attempts have been reported in constructing functional association predictors solely based on GO information. In some studies, associations between proteins in a pair are assessed in terms of the similar GO terms [29], while other studies evaluate functional associations based on either information content [30] or GO structural hierarchy [31]. In a recent study, GO annotations have been used to construct a PPI network for yeast by measuring similarity between two gene ontology terms with a relative specificity semantic relation [32].

Therefore, GO can be utilized as a useful informative resource to either predict or further analyze the predicted PPI datasets. However, ontology annotation is an incomplete process and suffers from inconsistency within and between genomes. In some cases, two confirmed interacting proteins are assigned two different GO annotations which are not equivalent in terms of information content. One protein is assigned a term that represents a broad type of activity, and its interacting partner is assigned a more specific term that represents a subtype of that activity. In other cases, some proteins have not even been assigned all three ontologies which make the interaction assessments more difficult without human intervention. Thus, the molecular functions of GO annotations of related proteins should be harmonized in relation to the information content and compared on a more general level. There are advantages and disadvantages associated with the harmonization of GO terms. The advantage is that the predicted relationship between proteins in a pair can be detected systematically using some keywords and it is not required to be verified manually. The disadvantage is that the integration of GO annotations and predicted PPIs might not be able to reveal the specific functions of the interacting proteins. However, knowing the fact that PPI prediction techniques are merely capable of specifying the general category of relationship between two proteins, this disadvantage is not a great source of concern.

In this study a global framework to refine computationally predicted datasets is developed. First, two experimental PPI datasets with high confidence were prepared for two model organisms *S. cerevisiae *and *C. elegans*. Assuming the experimentally confirmed pairs are true, the GO annotations of these interacting proteins were utilized to extract keywords which represent general category functions of the proteins. Then, a set of heuristic rules was established to be satisfied by the predicted interacting protein pairs based on extracted keywords and the fact that two proteins acting in the same cellular components are more likely to interact than those located in different components. Next, four computational methods representing four out of six categories of prediction techniques, mentioned earlier in this section, were selected. Using these methods, four predicted datasets were created for each organism of interest. The heuristic rules were applied to these predicted datasets. When a predicted pair of interacting proteins satisfied the rules it was considered a true positive, otherwise the pair was assumed false positive and removed from the dataset. The results show that the filtered datasets have higher true positive fractions than non-filtered datasets and the improvement is statistically significant.

## Results and Discussion

Using information deposited in the UNIPROT and GO databases, the experimentally obtained protein pairs for yeast and worm were processed, resulting in 1042 non-redundant GO term information (including 4391 yeast proteins) and 748 non-redundant GO term information (including 3390 worm proteins), respectively. These pieces of term information were further clustered, resulting in 35 and 25 keywords for yeast and worm, respectively (see Additional File 2).

### Significant keywords

Low frequency of appearance of some keywords in the training dataset indicates that all extracted keywords do not contribute equally to discriminate GO annotations. As listed in Table 1, the frequency of appearance of each keyword was ranked in descending order. The eight top-ranking keywords were chosen for the following analyses, and the remaining keywords (27 in yeast and 17 in worm) were grouped and called "RK". In order to evaluate the significance of these top-ranking keywords, the sensitivity and specificity analysis was conducted. Sensitivity (SN) is the percentage of protein pairs that are recovered using a certain keyword or a group of keywords when they are applied back to the source (the training dataset). Specificity (SP) is the percentage of protein pairs recovered when keywords are applied to predicted datasets (the test datasets). The sensitivity of each keyword was calculated as:

**Table 1:** Frequencies of keywords extracted from experimentally obtained yeast protein pairs

**Keywords**	**frequency**
Binding	3337
*ase *activity	2797
Porter activity	397
Transcription activity	372
Ribosome	134
Translation activity	58
Structural activity	51
Receptor activity	23
Remaining keywords (27 keywords)	230

SN of a keyword=number of pairs represented by the keywordtotal number of pairs in the training dataset×100=1x∑i=1xni×100
 MathType@MTEF@5@5@+=feaafiart1ev1aaatCvAUfKttLearuWrP9MDH5MBPbIqV92AaeXatLxBI9gBaebbnrfifHhDYfgasaacH8akY=wiFfYdH8Gipec8Eeeu0xXdbba9frFj0=OqFfea0dXdd9vqai=hGuQ8kuc9pgc9s8qqaq=dirpe0xb9q8qiLsFr0=vr0=vr0dc8meaabaqaciaacaGaaeqabaqabeGadaaakeaacqWGtbWucqWGobGtcqqGGaaicqWGVbWBcqWGMbGzcqqGGaaicqWGHbqycqqGGaaicqWGRbWAcqWGLbqzcqWG5bqEcqWG3bWDcqWGVbWBcqWGYbGCcqWGKbazcqGH9aqpdaWcaaqaaiabd6gaUjabdwha1jabd2gaTjabdkgaIjabdwgaLjabdkhaYjabbccaGiabd+gaVjabdAgaMjabbccaGiabdchaWjabdggaHjabdMgaPjabdkhaYjabdohaZjabbccaGiabdkhaYjabdwgaLjabdchaWjabdkhaYjabdwgaLjabdohaZjabdwgaLjabd6gaUjabdsha0jabdwgaLjabdsgaKjabbccaGiabdkgaIjabdMha5jabbccaGiabdsha0jabdIgaOjabdwgaLjabbccaGiabdUgaRjabdwgaLjabdMha5jabdEha3jabd+gaVjabdkhaYjabdsgaKbqaaiabdsha0jabd+gaVjabdsha0jabdggaHjabdYgaSjabbccaGiabd6gaUjabdwha1jabd2gaTjabdkgaIjabdwgaLjabdkhaYjabbccaGiabd+gaVjabdAgaMjabbccaGiabdchaWjabdggaHjabdMgaPjabdkhaYjabdohaZjabbccaGiabdMgaPjabd6gaUjabbccaGiabdsha0jabdIgaOjabdwgaLjabbccaGiabdsha0jabdkhaYjabdggaHjabdMgaPjabd6gaUjabdMgaPjabd6gaUjabdEgaNjabbccaGiabdsgaKjabdggaHjabdsha0jabdggaHjabdohaZjabdwgaLjabdsha0baacqGHxdaTcqaIXaqmcqaIWaamcqaIWaamcqGH9aqpdaWcaaqaaiabigdaXaqaaiabdIha4baadaaeWbqaaiabd6gaUnaaBaaaleaacqWGPbqAaeqaaaqaaiabdMgaPjabg2da9iabigdaXaqaaiabdIha4bqdcqGHris5aOGaey41aqRaeGymaeJaeGimaaJaeGimaadaaa@C7A0@

where *x *is the total number of pairs in the experimental dataset (the training dataset). If *n*_*i *_= 1, it indicates that two proteins in pair *i *are represented by a keyword; and *n*_*i *_= 0, otherwise. Cumulative sensitivity of all keywords was obtained as:

Cumulative SN=1x∑i=1x∑j=1znij×100
 MathType@MTEF@5@5@+=feaafiart1ev1aaatCvAUfKttLearuWrP9MDH5MBPbIqV92AaeXatLxBI9gBaebbnrfifHhDYfgasaacH8akY=wiFfYdH8Gipec8Eeeu0xXdbba9frFj0=OqFfea0dXdd9vqai=hGuQ8kuc9pgc9s8qqaq=dirpe0xb9q8qiLsFr0=vr0=vr0dc8meaabaqaciaacaGaaeqabaqabeGadaaakeaacqWGdbWqcqWG1bqDcqWGTbqBcqWG1bqDcqWGSbaBcqWGHbqycqWG0baDcqWGPbqAcqWG2bGDcqWGLbqzcqqGGaaicqWGtbWucqWGobGtcqGH9aqpdaWcaaqaaiabigdaXaqaaiabdIha4baadaaeWbqaamaaqahabaGaemOBa42aaSbaaSqaaiabdMgaPjabdQgaQbqabaaabaGaemOAaOMaeyypa0JaeGymaedabaGaemOEaOhaniabggHiLdaaleaacqWGPbqAcqGH9aqpcqaIXaqmaeaacqWG4baEa0GaeyyeIuoakiabgEna0kabigdaXiabicdaWiabicdaWaaa@5827@

where *z *is the number of keywords. If *n*_*ij *_= 1, it shows that two proteins in pair *i *are represented by the common keyword *j*; and *n*_*ij *_= 0, otherwise. Cumulative sensitivity demonstrates the recovery power of all keywords collectively when they are applied to the source (training set). Specificity of a keyword and cumulative specificity of all keywords are similarly defined and calculated:

SP of a keyword=number of pairs represented by the keywordtotal number of pairs in the test set×100=1y∑i=1yni×100
 MathType@MTEF@5@5@+=feaafiart1ev1aaatCvAUfKttLearuWrP9MDH5MBPbIqV92AaeXatLxBI9gBaebbnrfifHhDYfgasaacH8akY=wiFfYdH8Gipec8Eeeu0xXdbba9frFj0=OqFfea0dXdd9vqai=hGuQ8kuc9pgc9s8qqaq=dirpe0xb9q8qiLsFr0=vr0=vr0dc8meaabaqaciaacaGaaeqabaqabeGadaaakeaacqWGtbWucqWGqbaucqqGGaaicqWGVbWBcqWGMbGzcqqGGaaicqWGHbqycqqGGaaicqWGRbWAcqWGLbqzcqWG5bqEcqWG3bWDcqWGVbWBcqWGYbGCcqWGKbazcqGH9aqpdaWcaaqaaiabd6gaUjabdwha1jabd2gaTjabdkgaIjabdwgaLjabdkhaYjabbccaGiabd+gaVjabdAgaMjabbccaGiabdchaWjabdggaHjabdMgaPjabdkhaYjabdohaZjabbccaGiabdkhaYjabdwgaLjabdchaWjabdkhaYjabdwgaLjabdohaZjabdwgaLjabd6gaUjabdsha0jabdwgaLjabdsgaKjabbccaGiabdkgaIjabdMha5jabbccaGiabdsha0jabdIgaOjabdwgaLjabbccaGiabdUgaRjabdwgaLjabdMha5jabdEha3jabd+gaVjabdkhaYjabdsgaKbqaaiabdsha0jabd+gaVjabdsha0jabdggaHjabdYgaSjabbccaGiabd6gaUjabdwha1jabd2gaTjabdkgaIjabdwgaLjabdkhaYjabbccaGiabd+gaVjabdAgaMjabbccaGiabdchaWjabdggaHjabdMgaPjabdkhaYjabdohaZjabbccaGiabdMgaPjabd6gaUjabbccaGiabdsha0jabdIgaOjabdwgaLjabbccaGiabdsha0jabdwgaLjabdohaZjabdsha0jabbccaGiabdohaZjabdwgaLjabdsha0baacqGHxdaTcqaIXaqmcqaIWaamcqaIWaamcqGH9aqpdaWcaaqaaiabigdaXaqaaiabdMha5baadaaeWbqaaiabd6gaUnaaBaaaleaacqWGPbqAaeqaaaqaaiabdMgaPjabg2da9iabigdaXaqaaiabdMha5bqdcqGHris5aOGaey41aqRaeGymaeJaeGimaaJaeGimaadaaa@BCF4@

Cumulative SP=1y∑i=1y∑j=1znij×100
 MathType@MTEF@5@5@+=feaafiart1ev1aaatCvAUfKttLearuWrP9MDH5MBPbIqV92AaeXatLxBI9gBaebbnrfifHhDYfgasaacH8akY=wiFfYdH8Gipec8Eeeu0xXdbba9frFj0=OqFfea0dXdd9vqai=hGuQ8kuc9pgc9s8qqaq=dirpe0xb9q8qiLsFr0=vr0=vr0dc8meaabaqaciaacaGaaeqabaqabeGadaaakeaacqWGdbWqcqWG1bqDcqWGTbqBcqWG1bqDcqWGSbaBcqWGHbqycqWG0baDcqWGPbqAcqWG2bGDcqWGLbqzcqqGGaaicqWGtbWucqWGqbaucqGH9aqpdaWcaaqaaiabigdaXaqaaiabdMha5baadaaeWbqaamaaqahabaGaemOBa42aaSbaaSqaaiabdMgaPjabdQgaQbqabaaabaGaemOAaOMaeyypa0JaeGymaedabaGaemOEaOhaniabggHiLdaaleaacqWGPbqAcqGH9aqpcqaIXaqmaeaacqWG5bqEa0GaeyyeIuoakiabgEna0kabigdaXiabicdaWiabicdaWaaa@582F@

where *y *is the total number of pairs in the predicted dataset (the test dataset). Cumulative specificity translates into the recovery power of all keywords when they are applied to a predicted dataset (test set).

**Figure 1:**
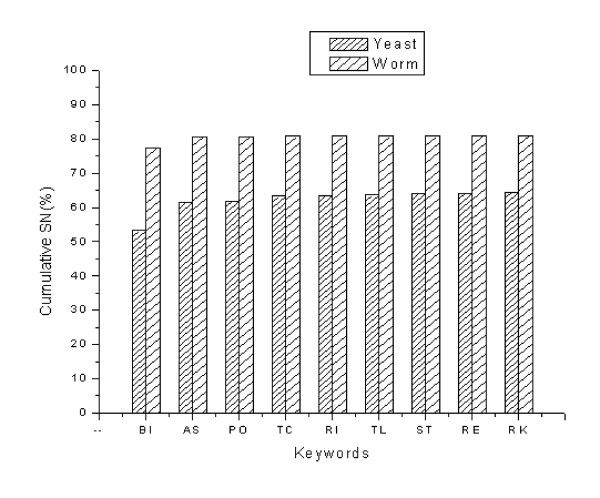
Cumulative sensitivity of keywords for yeast and worm. Each column indicates the sensitivity of a keyword in addition to the sensitivities of previous keywords. The highest sensitivities are 64.43% and 80.88% in the yeast and worm training datasets, respectively. Abbreviations for keywords are as follows: BI (binding), AS (ase activity), PO (porter activity), TC (transcription activity), RI (ribosome), TL (translation activity), ST (structural activity), RE (receptor activity), and RK (remaining keywords).

Figure 1 illustrates the cumulative sensitivity variations among extracted keywords in both studied organisms. The cumulative sensitivity of all 35 yeast keywords is 64.43%. When only the top 8 high-scored keywords are considered, the cumulative sensitivity is 64.21%, indicating that the remaining keywords imposed relatively insignificant contribution to the cumulative sensitivity. Similarly, in the worm dataset the same eight keywords contributed to 80.83% cumulative sensitivity and the remaining keywords increased that value to 80.88% (i.e. 0.05% increase). Thus, in trade-off between the lowest number of keywords and the highest cumulative sensitivity, it is favourable to neglect 27 keywords in yeast (17 keywords in worm) with the cost of only 0.22% (0.05% in worm) lower sensitivity.

**Figure 2:**
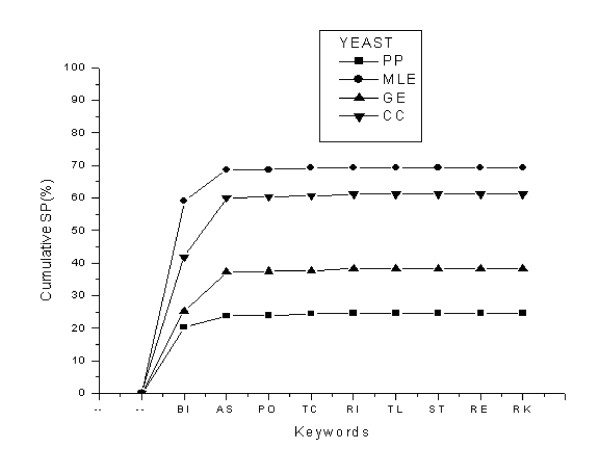
Cumulative specificity of trained keywords, applied to the four predicted PPI datasets in yeast. Each data point indicates the specificity of a keyword in addition to the specificities of previous keywords. Abbreviations for keywords are as follows: BI (binding), AS (ase activity), PO (porter activity), TC (transcription activity), RI (ribosome), TL (translation activity), ST (structural activity), RE (receptor activity), and RK (remaining keywords). RK includes 27 keywords with negligible contribution to cumulative SP.

**Figure 3:**
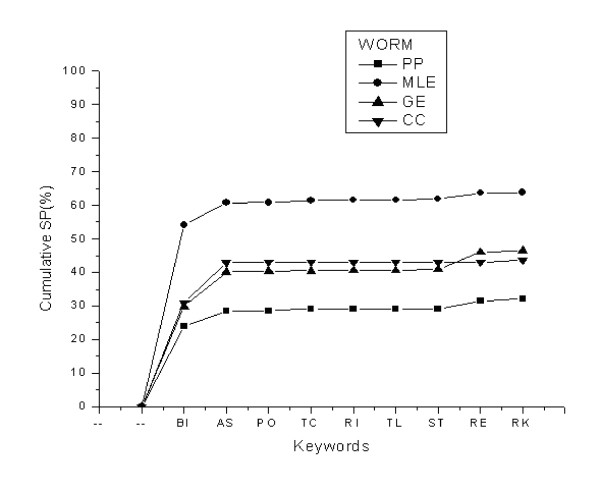
Cumulative specificity of trained keywords, applied to the four predicted PPI datasets in worm. Each data point indicates the specificity of a keyword in addition to the specificities of previous keywords. Abbreviations for keywords are as follows: BI (binding), AS (ase activity), PO (porter activity), TC (transcription activity), RI (ribosome), TL (translation activity), ST (structural activity), RE (receptor activity), and RK (remaining keywords). RK includes 17 keywords with negligible contribution to cumulative SP.

In order to further examine the significance of the extracted top-ranking keywords from the training dataset, the cumulative specificities of the keywords applied to four predicted protein-protein interaction datasets were calculated. These four predicted datasets were obtained using computational methods including phylogenetic profiles (PP), gene expression (GE), maximum likelihood estimation (MLE), and chance co-occurrence distribution (CC). The implementation of these methods is described in Additional File 1. As illustrated in Figure 2, the cumulative specificity of yeast varies from 25% in PP dataset to 69% in MLE dataset. In all four predicted datasets specificity changes very slightly when it is extended from eight top-ranking keywords to all extracted keywords. Similarly, in Figure 3, the worm dataset cumulative specificity ranges from 32% in the PP dataset to 64% in the MLE dataset using the eight top-ranking keywords. The remaining keywords exert negligible changes to the cumulative specificities in all four datasets. Therefore, these top-ranking eight keywords extracted from the experimental datasets of both organisms are capable of representing the common functions of interacting proteins either experimentally specified or computationally predicted.

Although the eight top-ranking keywords significantly recover the experimental or predicted datasets, the cumulative sensitivity or specificity is not distributed equally as seen in Figures 1, 2, 3. Among the keywords "binding" (BI) is an exception with the sensitivity of 53.22% in the yeast dataset, for instance, compared to 8.20% for "ase activity" (AS), 0.43% for "porter activity" (PO), and so on. This drastic difference between the sensitivity or specificity of this particular keyword and that of other keywords stems from the fact that our experimental datasets are collections of protein interactions detected mainly by the two-hybrid technique. This high-throughput technique detects physical interactions among proteins in which binding of a protein to the active site of another protein is a crucial step. Accordingly, most of these protein pairs are assigned the "binding" molecular function annotation in GO database. On the other hand, the contribution of keywords such as "receptor activity" (RE) in the cumulative sensitivity is 0.20% which is not a remarkable contribution; however, it is significant when it is compared with 0.22% increase in cumulative sensitivity by "remaining keywords" (RK) which represents 27 keywords in the case of yeast.

It should be noted that the highest obtainable cumulative sensitivity, in yeast for example, is 64.43% by means of all keywords and 64.21% by means of eight top-ranking keywords. Currently, it is impossible to obtain complete sensitivity (100%), as some experimental pairs do not have consistent annotations. This inconsistency comes from the fact that there are deficiencies in either annotation or experimental techniques. In case of the worm dataset the inconsistency is worse. Only 55% of worm genes are annotated and many annotations are not consistent. It is also notable that the GO molecular function annotations can not be used directly as keywords. When the definitions of the GO molecular functions were considered as keywords, the cumulative sensitivity of the training dataset was only 45.00%, comparing to that of 64.43% when the keyword extraction approach was implemented.

### Heuristic Rules

Protein interactions take place in either permanent or transient complexes formed in a cell, suggesting that proteins are required to exist in close proximity to interact physically [33]. Hence, the concept of protein-protein interactions in cellular systems is based on the following two observations: (i) interacting proteins often perform similar general functions, assuming that two proteins functioning in the same general category are more likely to interact than two proteins involved in different functions: (ii) co-localization may serve as an useful tool to predict protein interactions. Physical interactions occur when two proteins are located in the same cellular component, either a permanent cellular location or a transient complex. Motivated by the two observations, two heuristic rules were set to be satisfied by predicted interacting protein pairs. These rules are:

(I) Two predicted proteins in the pair should match one of the eight trained function keywords.

(II) Two predicted proteins in the pair should be in the same GO cellular components.

As many computational protein interaction prediction techniques suffer from mass false positive predictions, satisfying the rules filters the predicted datasets and removes the false interactions to some extent.

**Figure 4:**
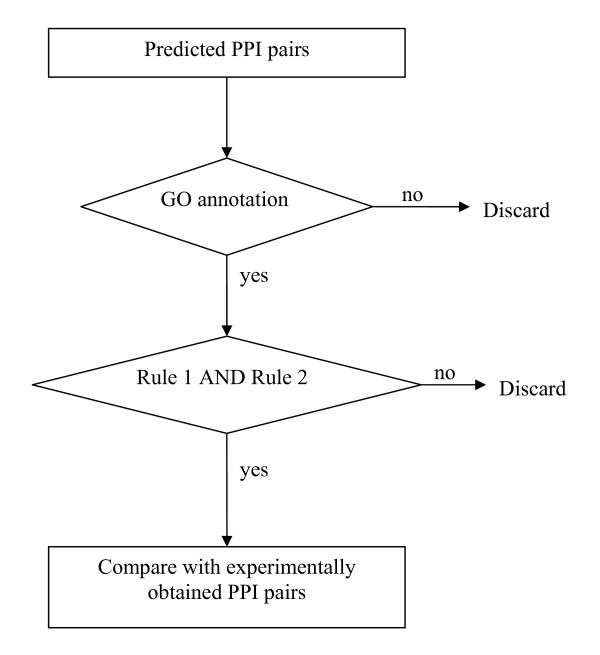
The flowchart of proposed algorithm to filter predicted PPI pairs.

Based on the algorithm depicted in Figure 4, these two rules were applied to the four predicted PPI datasets for each of the studied organisms. The algorithm reads PPI pairs predicted by PP, GE, MLE, and CC sequentially. It then examines if two proteins in the same pair possess the GO annotations: molecular function and cellular component. If so, such a pair with annotations is checked with the proposed rules. Satisfying rule I and rule II, this protein pair is considered as an interacting one. Finally, the filtered predicted dataset is compared with experimental dataset to assess the level of agreement with experimental results.

### Statistical analysis

In order to evaluate the improvement made by applying rules to the predicted PPI datasets, the signal-to-noise ratio (SNR) [34] was employed. SNR is a measure of signal strength relative to background noise. In bioinformatics, SNR is translated to the ratio of the capability of a computational method in creating protein pairs to pairing proteins on a random basis. Therefore, we define SNR as the ratio of the true positive fraction of a predicted dataset to the true positive fraction of a randomly selected dataset with the same sample size. True positive fraction of a dataset is the ratio of matched protein pairs with the experimental dataset to the total number of pairs in the same dataset:

SNR=(matched pairs/total pairs)predicted dataset(matched pairs/total pairs)random dataset
 MathType@MTEF@5@5@+=feaafiart1ev1aaatCvAUfKttLearuWrP9MDH5MBPbIqV92AaeXatLxBI9gBaebbnrfifHhDYfgasaacH8akY=wiFfYdH8Gipec8Eeeu0xXdbba9frFj0=OqFfea0dXdd9vqai=hGuQ8kuc9pgc9s8qqaq=dirpe0xb9q8qiLsFr0=vr0=vr0dc8meaabaqaciaacaGaaeqabaqabeGadaaakeaacqWGtbWucqWGobGtcqWGsbGucqGH9aqpdaWcaaqaaiabcIcaOiabd2gaTjabdggaHjabdsha0jabdogaJjabdIgaOjabdwgaLjabdsgaKjabbccaGiabdchaWjabdggaHjabdMgaPjabdkhaYjabdohaZjabc+caViabdsha0jabd+gaVjabdsha0jabdggaHjabdYgaSjabbccaGiabdchaWjabdggaHjabdMgaPjabdkhaYjabdohaZjabcMcaPmaaBaaaleaacqWGWbaCcqWGYbGCcqWGLbqzcqWGKbazcqWGPbqAcqWGJbWycqWG0baDcqWGLbqzcqWGKbazcqqGGaaicqWGKbazcqWGHbqycqWG0baDcqWGHbqycqWGZbWCcqWGLbqzcqWG0baDaeqaaaGcbaGaeiikaGIaemyBa0MaemyyaeMaemiDaqNaem4yamMaemiAaGMaemyzauMaemizaqMaeeiiaaIaemiCaaNaemyyaeMaemyAaKMaemOCaiNaem4CamNaei4la8IaemiDaqNaem4Ba8MaemiDaqNaemyyaeMaemiBaWMaeeiiaaIaemiCaaNaemyyaeMaemyAaKMaemOCaiNaem4CamNaeiykaKYaaSbaaSqaaiabdkhaYjabdggaHjabd6gaUjabdsgaKjabd+gaVjabd2gaTjabbccaGiabdsgaKjabdggaHjabdsha0jabdggaHjabdohaZjabdwgaLjabdsha0bqabaaaaaaa@9F72@

SNR was calculated for all four predicted datasets for each of yeast and worm in the following two circumstances: before applying the rules to a dataset (raw dataset), and after applying the rules to a dataset (filtered dataset). The effect of the rules on the reduction of false positive predictions was measured by the *strength *(*S*):

S=SNRFiltered DatasetSNRRaw Dataset
 MathType@MTEF@5@5@+=feaafiart1ev1aaatCvAUfKttLearuWrP9MDH5MBPbIqV92AaeXatLxBI9gBaebbnrfifHhDYfgasaacH8akY=wiFfYdH8Gipec8Eeeu0xXdbba9frFj0=OqFfea0dXdd9vqai=hGuQ8kuc9pgc9s8qqaq=dirpe0xb9q8qiLsFr0=vr0=vr0dc8meaabaqaciaacaGaaeqabaqabeGadaaakeaacqWGtbWucqGH9aqpdaWcaaqaaiabdofatjabd6eaojabdkfasnaaBaaaleaacqWGgbGrcqWGPbqAcqWGSbaBcqWG0baDcqWGLbqzcqWGYbGCcqWGLbqzcqWGKbazcqqGGaaicqWGebarcqWGHbqycqWG0baDcqWGHbqycqWGZbWCcqWGLbqzcqWG0baDaeqaaaGcbaGaem4uamLaemOta4KaemOuai1aaSbaaSqaaiabdkfasjabdggaHjabdEha3jabbccaGiabdseaejabdggaHjabdsha0jabdggaHjabdohaZjabdwgaLjabdsha0bqabaaaaaaa@590E@

As seen in Table 2, SNR values for all filtered data were larger than those for corresponding raw data, indicating that the proposed algorithm can reduce false positive prediction of PPI pairs. Depending on the PPI-predicting method employed, the *S *value varies from 2.32 to 19.90 for the yeast datasets, and 1.96 to 3.94 for the worm datasets, implying that the proposed algorithm exerts a stronger influence on improving PPI pairs predicted by the PP method than those predicted by the MLE method. In other words, the MLE approach predicts more robust protein pairs than other three methods used in this study.

**Table 2:** Comparison of SNR and S value of predicted datasets before (raw data) and after (filtered data) removing false positive protein-protein interaction pairs

	**Yeast**			**Worm**		
**Method**	SNR* (raw data)	SNR* (filtered data)	**S**	SNR* (raw data)	SNR* (filtered data)	**S**

PP	1.59	15.78	9.90	32.78	129.0	3.94
GE	1.89	8.83	4.67	27.36	66.0	2.41
CC	3.10	12.21	3.94	51.88	202.0	3.89
MLE	13.44	31.14	2.32	197.2	387.0	1.96

*SNR was calculated based on Equation (7). The size of the random dataset was the same as that of protein pairs predicted by each respective method and their true positive fractions were obtained using the mean of 100 trials.

Given the fact that protein interactions are required for cell functioning and permanent or transient interactions between proteins are regulated under biological circumstances, this statistical analysis can well distinguish between the predicted/filtered datasets and random selection of interaction. The predicted and filtered set of interactions in a dataset is significant when the true positive fraction of the dataset is greater than that in a randomly selected dataset. In other words, the selection of interacting proteins is significant when the strength value is greater than 1.

The algorithm proposed here to reduce the number of false positive predicted protein pairs could also be extended to evaluate and compare the effectiveness of PPI prediction among different computational approaches. The algorithm is a post-prediction processing step that is applied to the resulted predicted dataset when a computational method is implemented. Thus, it can be attached to any computational approach for further analysis of predicted results. However, it should be noted that ontology is an ongoing process. With more genes assigned with GO terms, the proposed filtering algorithm is a promising approach to reduce the number of false positive interactions and thus to enhance the accuracy of PPI prediction.

### UNIPROT/GO limitations and intrinsic deviation of the analysis

UNIPROT and GO employ different strategies to annotate proteins. In cases where experimental information is not available, sequence homology is the strategy that is used to assign biochemical information to proteins. Thus, there is a concern that protein pairs detected by the proposed algorithm as true positives may also be inferred by homology which consequently demonstrates low accuracy of filtered protein pairs. We compared our filtered datasets with interacting proteins reported in KOG database provided by NCBI. This database includes orthologous and paralogous proteins of eukaryotic species. Each group is associated with a conserved and specific function. Our examination shows that, on average, only 1.24% of the protein pairs that satisfies the rules can be predicted through homology. Refer to Additional File 1 for more information.

Annotation is an ongoing process and there are many proteins: i) to be annotated, ii) erroneously annotated, iii) annotated but do not comply with experimental findings, iv) with unidentified locations and ubiquitous status. These proteins and their pair-wise interactions with other proteins contribute to the intrinsic deviation of the proposed approach to filtering false positive predictions. To confine the bias caused by the above mentioned reasons and decrease the deviation of the resulted filtered datasets, the rules have been applied solely to those protein pairs whose GO annotations are available. Furthermore, protein pairs with GO annotations suffer from inconsistency among annotations. Extracted functional keywords address this deficiency and can recover proteins pairs whose general functions are similar, even though their GO annotations are not in the same level of information content. Nevertheless, the deviation resulted from this inconsistency can be well represented by means of sensitivity and specificity. For example, in Yeast high confidence experimental dataset (training set), there are 16507 protein pairs of which 15748 protein pairs (95%) contain GO annotation. This figure indicates that the coverage of GO annotations for well-studied organisms is satisfactory. Of 15748 protein pairs with annotations, 10146 pairs contain similar function annotations accounting for 64.43% which is equal to the sensitivity of the extracted keywords.

The intrinsic deviation partially depends on the precision of annotation process. In fact, annotation process has shifted from manual annotation to automated annotation due to growing influx of protein information in the past few years and time-consuming process of manual curation. Shifting from manual annotation to automated procedures decreased the accuracy of annotation process. However, with the aid of powerful text-mining tools the accuracy of annotation is now satisfactory. Based on an investigation in 2002, Incellico Inc. reported that the accuracy of GO annotation is 95% [49]. GOA is a project aiming at providing high quality GO annotation to proteins in UNIPROT knowledgebase [35]. Camon *et al*. [36] evaluated the GO annotation retrieval of GOA project and reported 91% precision. Recently, Couto *et al*. [37] presented a text-mining technique with 93% precision in annotation.

## Conclusion

Gene ontology annotation was used as a common ground to evaluate protein pairs predicted by four different PPI-predicting methods. Molecular function annotations in the Gene Ontology database were used to extract discriminating keywords, upon which heuristic rules were set. The rules were incorporated into an algorithm by which predicted datasets were filtered and false positive predictions were partially removed from the datasets. When only eight top-ranking keywords were chosen, over 99% of molecular function could be recovered as indicated by the cumulative sensitivity for both experimentally obtained and computationally predicted protein pairs. The effectiveness of the proposed algorithm to filter false positive predicted protein pairs varies from one method to another. The proposed algorithm is unbiased and could be implemented on all computational methods to increase the accuracy of PPI prediction. As more genes are assigned with GO annotations, the proposed filtering algorithm will become even more effective.

## Methods

### Experimental datasets

The dataset containing experimentally obtained protein pairs was used to extract the functional keywords from the GO annotations. The yeast dataset was compiled from the following three sources: (1) von Mering *et al*. [38] reported high confident yeast protein pairs that were confirmed by at least two experimental methods, resulting in 1920 protein pairs; (2) the BIND database [39] contains 10618 yeast protein pairs that were experimentally confirmed and manually curated; and (3) CYGD [40] contains 10472 experimentally verified yeast protein pairs. Combining these three sources resulted in 16507 non-duplicated yeast protein pairs, consisting of 4391 proteins.

The worm dataset was constructed from BIND [39] and Li *et al*., [41]. They reported 4960 and 6629 protein pairs, respectively. These pairs were obtained by means of the yeast two-hybrid technique and manually curated. After removing duplicated pairs the dataset consists of 7081 pairs, comprising 3390 proteins in *C. elegans*.

### Computational protein-protein interaction methods

Four PPI predicting methods from four out of six categories discussed in the Introduction section were chosen, including phylogenetic profiles (PP), chance co-occurrence distribution coefficient (CC), gene expression profiles (GE), and maximum likelihood estimation (MLE). The criteria of choosing these methods were based on: their genome-wide applicability and competitive results in the category [42-45]. The implementation of these methods is presented in Additional File 1.

### Gene ontology and annotations

The GO annotations of proteins were retrieved from the UNIPROT knowledgebase [46] which is collaborated with the GO database [47]. Annotations in both the UNIPROT and GO databases are updated on a regular basis. In this study, the UNIPROT knowledgebase, Release 8 (June 2006) and the GO database, Version 1.362 (May 2006) were used to extract keywords for the false positive reduction on the predicted protein pairs.

### Keyword extraction

Proteins involved in experimentally verified protein pairs were submitted to UNIPROT. Then GO and InterPro cross-reference assignments of the protein were retrieved. Through "*interpro2go*" (retrieved from Mappings to GO on the GO website), all InterPro entries were mapped to GO terms and the GO terms of each protein were searched using AMIGO term search engine. The searched GO term information of each protein was collected and redundant information was removed. The remaining term definition relevant to the molecular function annotation (a part of term information) was compiled and used as a training dataset. The dataset was further manually grouped into different clusters according to their general molecular activities; for instance, GO:0003723 and GO:0000166 were placed in the same cluster because of molecule-binding activities. Refer to Additional File 2 for a complete listing of all clusters for *S. cerevisiae *and *C. elegans*.

In order to determine a representative keyword in a cluster, the number of occurrences (*n*) of a word in a cluster was counted, and the probability of finding that word in the training dataset was calculated using Poisson distribution:

p(n)=e−λλnn!
 MathType@MTEF@5@5@+=feaafiart1ev1aaatCvAUfKttLearuWrP9MDH5MBPbIqV92AaeXatLxBI9gBaebbnrfifHhDYfgasaacH8akY=wiFfYdH8Gipec8Eeeu0xXdbba9frFj0=OqFfea0dXdd9vqai=hGuQ8kuc9pgc9s8qqaq=dirpe0xb9q8qiLsFr0=vr0=vr0dc8meaabaqaciaacaGaaeqabaqabeGadaaakeaacqWGWbaCcqGGOaakcqWGUbGBcqGGPaqkcqGH9aqpcqWGLbqzdaahaaWcbeqaaiabgkHiTGGaciab=T7aSbaakmaalaaabaGae83UdW2aaWbaaSqabeaacqWGUbGBaaaakeaacqWGUbGBcqGGHaqiaaaaaa@3BEE@

where *λ *= *N*·*f*, in which *N *is the total number of words in a cluster, and *f *is the relative frequency of that word in the whole training dataset. To avoid floating point errors and facilitate computation, *n! *was approximated by Stirling's approximation, resulting in

ln *p*(*n*) = -*λ *+ *n *ln *λ *- *n *ln(*n*) + *n *- 1

This calculation is valid when the total number of words in the training dataset is much greater than *N *or when *f *is small. In order to identify most comprehensive words in each cluster, grammatical terms such as proposition, and chemical formulae were purposefully eliminated. In the "enzymatic function" cluster, all enzyme activities were considered as "*ase activity*" since enzymes are introduced with "*ase*" suffix in biochemistry literature. In each cluster the word with the most negative *ln p *value was selected as the representative keyword.

## Supplementary Material

Additional file 2 The complete listing of all GO molecular function clusters, their representing keywords, and corresponding *ln p *values for both studied organisms *S. cerevisiae*, and *C. elegans*. Click here for file

Additional file 1 Description of the implementation of the four selected computational protein-protein interaction prediction methods including phylogenetic profiles (PP), gene co-expression (GE), chance co-occurrence distribution coefficient (CC), and maximum likelihood estimation (MLE). Click here for file
