# Supplementary material for: False positive reduction in protein-protein interaction predictions using gene ontology annotations
Source: BMC Bioinformatics. 2007 Jul 23;8:262. doi: 10.1186/1471-2105-8-262 (PMC1941744; doi:10.1186/1471-2105-8-262)
Supplement: Additional file 1 — Description of the implementation of the four selected computational protein-protein interaction prediction methods including phylogenetic profiles (PP), gene co-expression (GE), chance co-occurrence distribution coefficient (CC), and maximum likelihood estimation (MLE). [file 1471-2105-8-262-S1.pdf]

Additional File 1

This file contains the detailed description of implementation of four selected computational methods to predict protein-protein interaction datasets. These methods are including phylogenetic profiles (PP), Gene co-expression profiles (GE), Chance co-occurrence probability distribution coefficient (CC), and Maximum likelihood estimation (MLE). Also the detail information on the comparison of predicted and filtered protein pairs with KOG protein pairs is presented in this file.

Phylogenetic profiles

The numbers of proteins studied in two organisms are: m=5863 in *S.cerevisiae* and m=12095 in *C.elegans*. The proteins of each organism were considered as queries and aligned against a database comprising 90 genomes using BLAST program. The list of reference genomes is in Table 1S. Genomes were obtained from www.ncbi.nlm.nih.gov.

Running BLAST program, using SEG filter over 75% similarity of the sequences, the output was a list of homolog proteins and their e-values within each genome that better match the query sequence. The best hit in each genome was taken as one bit in the profile and then profiles were created for each individual protein. These profiles should be converted into binary profiles in the form of 1 and 0 to represent the presence or absence of an individual protein in other genomes. To convert e-values to binary numbers it was required to know if the alignment score for each protein sequence P_i was statistically significant. Statistical significance of an alignment was described by the probability of finding a higher score when two sequences were compared based on a random selection. This probability depends on the number of comparisons made. If the number of proteins encoded in query genome is m and the number of encoded proteins in 90 reference genomes is p the total number of comparisons is: $m \times p$. Therefore, the probability of finding a match for an individual protein sequence is$1/(m \times p)$. In this study p=370461 and m for each organism is given above. We considered this probability as a threshold based on which e-values could be translated to present or absent status. Once the binary profiles were established, they were compared to find interacting proteins. Matching profiles were considered ‘interacting’.

Table 1S. *List of reference genomes employed in phylogenetic profiles approach*

A. fulgidus	*A. pernix*	*H. spNRC1*	*M. acetivorans*	*M. jannaschii*
M. kandleriAV19	*M. mazeiGoe1*	*M. thermautotrophicus*	*P. abyssi*	*P. aerophilum*
P. furiosusDSM3638	*P. horikoshii*	*S. solfataricus*	*S. tokodaii*	*T. acidophilum*
T. volcanium	*A. aeolicus*	*A. tumefaciens Cereon*	*A. tumefaciens UWash*	*B. burgdorferi*
B. halodurans	*B. melitensis*	*B. subtilis*	*B. spAPS*	*C. acetobutylicum ATCC824*
C. crescentusCB15	*C. glutamicum*	*C. jejuni*	*C. muridarum*	*C. perfringens*
C. pneumoniaeAR39	*C. pneumoniae*	*C. pneumoniaeJ138*	*C. trachomatis*	*D. radioduransR1*
E. coliK12	*E. coli O157H7*	*E. coli O157H7EDL933*	*F. nucleatum*	*H. influenzaeRDKW20*
H. pylori26695	*H. pyloriJ99*	*L. innocua*	*L. lactis*	*L. monocytogenes*
M. genitalium	*M. leprae*	*M. loti*	*M. pneumoniae*	*M. pulmonis*
M. tuberculosis CDC1551	*M. tuberculosis H37Rv*	*N. meningitidisMC58*	*N. meningitides Z2491*	*N. spPCC7120*
P. aeruginosa	*P. multocida*	*R. conorii*	*R. prowazekii*	*R. solanacearum*
S. aureusMW2	*S. aureusMu50*	*S. aureusN315*	*S. coelicolor*	*S. meliloti*
S. pneumoniaeR6	*S. pneumoniae*	*S. pyogenesM1GAS*	*S. pyogenesMGAS8232*	*S. typhi*
S. typhimuriumLT2	*S. spPCC6803*	*T. maritima*	*T. pallidum*	*T. tengcogensis*
U. urealyticum	*V. cholerae*	*X. citri*	*X. campestris*	*X. fastidiosa*
Y. pestis	*A. thaliana*	*C. elegans*	*D. melanogaster*	*E. cuniculi*
H. sapiens	*N. crassa*	*S. cerevisiae*	*S. pombe*	*C. briggsae*

Gene co-expression profiles

Genes with similar co-expression patterns are more likely to interact. To find out which genes are co-expressed, the expression levels of the studied genes were extracted from normalized DNA microarray data files obtained from Stanford Microarray Database (SMD). Each file corresponds to an experiment. All expression values were collected in a gene expression matrix in which each row represents a different gene and each column corresponds to a different microarray experiment (100 experiments in *S. cerevisiae*, 575 experiments in *C. elegans*). The matrix is supplied into EXPANDER program for

clustering. Choosing click algorithm to cluster genes, the results obtained for each organism is in Table 2S:

***Table 2S**. The results of clustering of proteins based on expression data using EXPANDER*

**organism**	**Number of clusters**	**Overall homogeneity**
S. cerevisiae	6	0.552
C. elegans	10	0.631

Genes in the same cluster are co-expressed genes in different biological conditions. These genes were paired and considered ‘interacting’.

Chance co-occurrence distribution

Genes with identical patterns of occurrence across organisms tend to prediction of interactions; however, the requirement that the profiles be identical restricts the number of links that can be established by such pylogenetic profiling. Thus, there is a technique that relies on scoring phylogenetic patterns and matches them based on those scores rather than identical profiles. The scoring function provides more information than the simple presence or absence of genes.

Chance co-occurrence probability distribution has been used as a measure of similarity between two phylogenetic profiles. Based on the probability that a given arbitrary degree of similarity between two profiles would occur by chance, with no biological pressure, the interaction predictions are drawn with the criterion used to reject the null hypothesis. The probability *P(z|N,x,y)* of observing by chance (i.e. no functional pressure) z co-occurrence of genes *X* and *Y* in a set of *N* genomes, given that *X* occurs in *x* genomes, and *Y* occurs in *y* genomes is calculated as follows:

$$P = \frac{w_z \overline{w}_z}{W} \tag{1S}$$

where w_z is the number of ways to distribute z co-occurrence over the N genomes, $\overline{w}_z$ is the number of ways of distributing x-z and y-z genes over the remaining N-z genomes, and W is the number of ways of distributing X and Y over N genomes without restriction. The final equation is as follows:

$$P = \frac{(N-x)!(N-y)!x!y!}{(x-z)!(y-z)!(N+z-x-y)!z!N!} \qquad (2S)$$

The general trend of –log(P) versus z for each protein pair (X,Y) is illustrated in Figure 1S. Critical co-occurrence, z_c, is defined as the minimum number of co-occurrences required between two proteins to be considered as interacting proteins. Thus, as shown in this figure, protein pairs whose –log(P) is higher than a cut-off threshold (here, 8) and $z \geq z_c$ were predicted as interacting proteins.

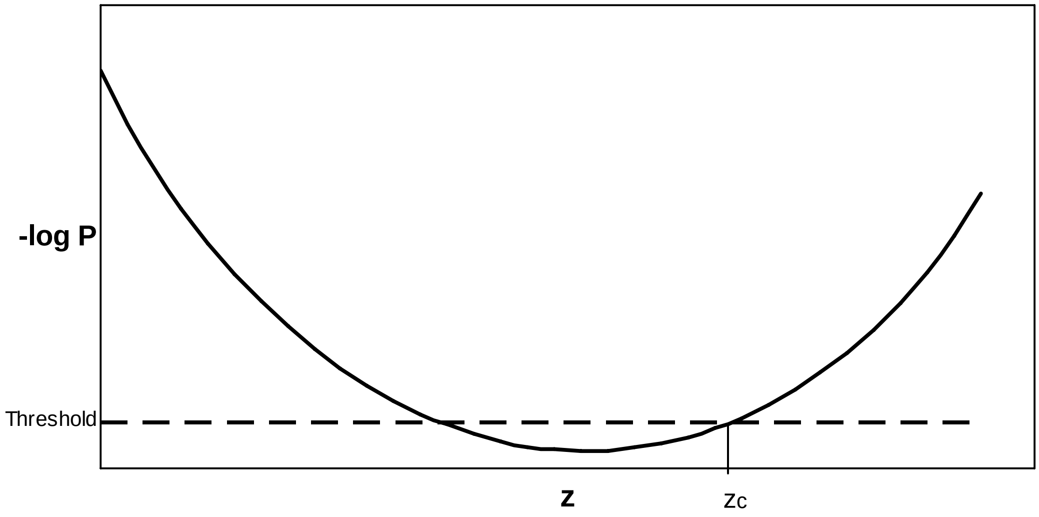

Figure 1S. *The negative logarithm of probability of z co-occurrence by chance (P) versus z. Based on the threshold value and* z_c *protein pairs with –log(P) located on the right-hand side portion of the curve are chosen as interacting proteins.*

Maximum Likelihood Estimation

The underlying hypothesis in this method is two proteins interact if and only if at least one pair of domains from the two proteins interact. Let D_1, D_2,….,D_M denote the M domains, and P_1, P_2,….P_N denote N proteins. P_{ij} denotes the protein pair of P_i and P_j, and D_{ij} denotes the domain pair of D_i and D_j. Treating protein-protein interactions, and domain-domain interactions as random variables, the probability of interacting two proteins under stated assumption is:

$$\Pr(P_{ij} = 1) = 1.0 - \prod_{D_{mn} \in P_{ij}} (1 - \lambda_{mn}) \tag{3S}$$

where λ_{mn}= Pr(D_{mn}=1) denotes the probability that domain D_m interacts with domain D_n. False positive rate (*fp*) and false negative rate (*fn*) are defined based on observed interactions. Let O_{ij} be the variable for the observed interaction result for proteins P_i and P_j. O_{ij} =1 if the interaction is observed and O_{ij}=0 otherwise. Then,

$$\begin{aligned} fn &= \Pr(O_{ij} = 0 \mid P_{ij} = 1) = 1.0 - \frac{\Pr(O_{ij} = 1, P_{ij} = 1)}{\Pr(P_{ij} = 1)} \geq 1.0 - \frac{\Pr(O_{ij} = 1)}{\Pr(P_{ij} = 1)} \\ &= 1.0 - \frac{number\, of\ observed\ pairs}{number\, of\ real\ \mathrm{int}eracting\ pairs} \end{aligned} \tag{4S}$$

$$\begin{aligned} fp &= \Pr(O_{ij} = 1 \mid P_{ij} = 0) = \frac{\Pr(O_{ij} = 1, P_{ij} = 0)}{\Pr(P_{ij} = 0)} \leq \frac{\Pr(O_{ij} = 1)}{\Pr(P_{ij} = 0)} \\ &= \frac{number\ of\ observed\ pairs}{total\ potential\ pairs - number\ of\ real\ \mathrm{int}eracting\ pairs} \end{aligned} \tag{5S}$$

Thus, the probability of observing a protein-protein interaction is:

$$\Pr(O_{ij} = 1) = \Pr(P_{ij} = 1)(1 - fn) + (1 - \Pr(P_{ij} = 1))\, fp \tag{6S}$$

The probability of the observed whole genome interaction dataset is

$$L = \prod (\Pr(O_{ij} = 1))^{O_{ij}} (1 - \Pr(O_{ij} = 1))^{1 - O_{ij}} \tag{7S}$$

where O_{ij}=1 if the interaction of P_i and P_j is observed and O_{ij}=0 otherwise. L is the likelihood and is a function of λ_{mn}, *fp,* and *fn*. In this calculation, *fn* and *fp* are determined based on Equations 4S and 5S as 0.437 and 9.6E-4 for yeast, and 0.883 and 9.7E-5 for worm, respectively. For example in case of yeast, the number of observed interactions (training set) is given as 16507 pairs. It is reported that in yeast proteome each protein interacts with approximately 5 proteins [45]. For 5863 yeast proteins in the proteome, it gives the number of real interactions of 29315 pairs. The total number of potential pairs is *m(m-1)/2* where *m* is 5863 proteins for yeast. Then, we compute λ_{mn} using a recursive formula. First, initial values for λ_{mn} are chosen. Then Pr(P_{ij}=1) and Pr(O_{ij}=1) are computed by equations (1S) and (4S), respectively. Parameter λ_{mn} is updated using the following equation

$$\lambda_{mn}^{(t)} = \frac{\lambda_{mn}^{(t-1)}}{N_{mn}} \sum_{i \in A_m, j \in A_n} \frac{(1-fn)^{O_{ij}} fn^{1-O_{ij}}}{\Pr(O_{ij} = o_{ij} \mid \lambda^{(t-1)})} \qquad (8S)$$

and likelihood function is computes by Equation (7S). Calculations continue until the value of likelihood function is unchanged within a certain error.

Comparison with homology

Homology-based protein-protein interactions rely on whole sequence alignment of primary structures and interactions are predicted when similarity between sequences is greater than a threshold E-value. To assess the extent of overlap between PPI pairs resulting from this study and those predicted by the homology-based alignment, KOG database was used. This database includes orthologous and paralogous proteins of eukaryotic species. Each group is associated with a conserved and specific function. Proteins in each group were considered interacting as they are assigned with similar functions and then interacting proteins were set in a pair-wise fashion. In case of yeast, our assembled dataset contains 8014 protein pairs involved in 1974 proteins in 2668 KOG groups. We compared this dataset with our predicted and filtered datasets, and observed that only 1.24% of the PPI pairs in the filtered dataset (see table below) existed in the KOG database. This means that approximately 99% of predicted and filtered PPI pairs are irrelevant to the homology-based alignment method using BLAST.

**Method**	**Total number of interactions in the filtered dataset**	**Number of interactions obtainable by homology in the filtered dataset**	**%**
CC	54935	1014	1.85
PP	27412	469	1.71
GE	92087	358	0.39
MLE	89024	880	0.99
		**Average**	1.24
